# Validation of the Framingham hypertension risk score in a middle eastern population: Tehran lipid and glucose study (TLGS)

**DOI:** 10.1186/s12889-021-10760-6

**Published:** 2021-04-24

**Authors:** Fatemeh Koohi, Ewout W. Steyerberg, Leila Cheraghi, Alireza Abdshah, Fereidoun Azizi, Davood Khalili

**Affiliations:** 1 Student Research Committee, Department of Epidemiology, School of Public Health and Safety, Shahid Beheshti University of Medical Sciences, Tehran, Iran; 2 Department of Epidemiology and Biostatistics, Research Institute for Endocrine Sciences, Shahid Beheshti University of Medical Sciences, Tehran, Iran; 3 Department of Biomedical Data Sciences, Leiden University Medical Centre, Leiden, The Netherlands; 4 School of Medicine, Tehran University of Medical Sciences, Tehran, Iran; 5 Endocrine Research Center, Research Institute for Endocrine Sciences, Shahid Beheshti University of Medical Sciences, Tehran, Iran; 6 Prevention of Metabolic Disorders Research Center, Research Institute for Endocrine Sciences, Shahid Beheshti University of Medical Sciences, Tehran, Iran

**Keywords:** Hypertension, Blood pressure, Risk prediction, Risk score, Primary prevention

## Abstract

**Background:**

The Framingham hypertension risk score is a well-known and simple model for predicting hypertension in adults. In the current study, we aimed to assess the predictive ability of this model in a Middle Eastern population.

**Methods:**

We studied 5423 participants, aged 20–69 years, without hypertension, who participated in two consecutive examination cycles of the Tehran Lipid and Glucose Study (TLGS). We assessed discrimination based on Harrell’s concordance statistic (c-index) and calibration (graphical comparison of predicted vs. observed). We evaluated the original, recalibrated (for intercept and slope), and revised (for beta coefficients) models.

**Results:**

Over the 3-year follow-up period, 319 participants developed hypertension. The Framingham hypertension risk score performed well in discriminating between individuals who developed hypertension and those who did not (c-index = 0.81, 95% CI: 0.79–0.83). Initially, there was a systematic underestimation of the original risk score (events predicted), which was readily corrected by a simple model revision.

**Conclusions:**

The revised Framingham hypertension risk score can be used as a screening tool in public health and clinical practice to facilitate the targeting of preventive interventions in high-risk Middle Eastern people.

**Supplementary Information:**

The online version contains supplementary material available at 10.1186/s12889-021-10760-6.

## Introduction

Hypertension is a major global health issue due to its high prevalence and importance as a modifiable risk factor for cardiovascular disease and premature mortality all over the world [1]. It may be asymptomatic up to the occurrence of clinical complications [2] and is also hard to manage effectively because of the lack of awareness and adherence to the treatment [3, 4].

According to global burden of disease (GBD) 2017, high systolic blood pressure (SBP) is the first leading risk factor for early death and disability, accounting for 10.4 million deaths and 218 million DALYs [5]. The number of people with raised blood pressure has increased worldwide, mainly in low- and middle-income countries [6]. Factors including population growth, aging, and behavioral risk factors, such as unhealthy diet, tobacco use, lack of physical activity, excess weight, and exposure to persistent stress, are attributable to the growing prevalence of hypertension [7]. Hence, one of the global non-communicable disease (NCD) targets, adapted by the World Health Assembly in 2013, is a 25% reduction in the prevalence of high blood pressure, defined as systolic blood pressure ≥ 140 mL/Hg and diastolic blood pressure ≥ 90 mL/Hg, by 2025 [8].

Evidence has shown that the risk of hypertension incidence depends on some clinical factors such as blood pressure, age, and BMI [9–11]. Therefore, an individual approach, based on risk stratification and targeted treatment of non-hypertension people who are at high risk for high blood pressure, may be more desirable [12], which requires a simple tool based on the prediction model. To apply such a model, ideally, the model has to be based on demographic and medical variables that are easily plain and available to non-specialized individuals and health care providers [13]. Thus, a risk assessment tool would be useful to identify high-risk individuals who should be targeted for early interventions to prevent or postpone the development of hypertension. Such models have potential public health implications and clinical applications in the prevention of hypertension [14].

Accordingly, several models to predict the risk of new-onset hypertension have been developed in different populations [12, 13, 15–20]. Framingham hypertension risk score is a well-known and straightforward model for predicting hypertension in adults; it includes only seven simple factors and, with a c-statistic of 0.788, has a good performance in estimating the 4-year risk of developing hypertension among participants in the Framingham study. However, further testing beyond the cohort in which the risk score was developed is necessary before its implementation in a new population [21]. We aimed to assess the predictive ability of the Framingham hypertension risk score in a Middle Eastern population-based cohort study.

## Methods

### Study design and population

The Tehran Lipid and Glucose Study (TLGS) is a population-based prospective study consisting of 15,005 participants, aimed to estimate the prevalence and incidence of NCDs [22]; the target population is a representative sample of an urban Iranian population, aged 3 to 69 years, living in Tehran, district No.13. The first examination cycle of the study started in 1999–2001 and after that, follow-up examinations have been repeated approximately every 3 years. Detailed description of rationale, design, and methodology of this study have been published [23]. Only the participants who attended both the 5th and 6th consecutive examination cycles were included. We excluded participants younger than 20 or older than 69 years old (*n* = 1181), had prevalent hypertension (*n* = 2004), had prevalent cardiovascular disease (*n* = 278) or serum creatinine values> 2 mg/dL (*n* = 1), had prevalent diabetes mellitus (*n* = 439), or had missing covariates (*n* = 1495) at examination cycle 5. After applying the exclusion criteria, 5423 individuals remained eligible for the current analysis.

### Assessment of hypertension

Systolic blood pressure (SBP) and diastolic blood pressure (DBP) were measured following standardized protocols at each TLGS examination cycle [24]. After resting for 15 min in sitting position, SBP and DBP were measured twice at the one-minute interval with a standard mercury sphygmomanometer calibrated by the Iranian Institute of Standards and Industrial Researches. The average of the two measures was taken as the systolic and diastolic blood pressures [22, 23].

Hypertension was defined as the SBP ≥ 140 mmHg or DBP ≥ 90 mmHg or the use of antihypertensive medications. We determined the incidence of hypertension by the presence of hypertension at examination cycle 6, among participants free of this condition at examination cycle 5 (Table 1).

**Table 1:** Baseline Characteristics of the Participants

Characteristic	Baseline Population*
No. of participants	5423
Mean age (SD), y	38.7 (11.7)
Women, n (%)	3067 (56.6)
Systolic blood pressure (SD), mm Hg	108.8 (11.8)
Diastolic blood pressure (SD), mm Hg	73.8 (7.9)
Current smoker, n (%)	772 (14.2)
Parental hypertension, n (%)	1399 (25.8)
Mean body mass index (SD), kg/m2	26.9 (4.7)

*SD* Standard Deviation

*Numbers represent mean ± SD for continuous variables and percentages are corresponding to “Yes” for dichotomous variables

### Assessment of covariates

Weight was measured in minimal clothes and without shoes on an electronic scale, which was placed on a flat surface and calibrated to zero before measurement. Height was measured in a standing position and without shoes using a tape meter. Body mass index was calculated as “weight (kilograms)/height (meters) squared”. Current smoking, prevalent cardiovascular diseases and parental hypertension were self-reported. Current smokers were defined as a person who smokes cigarettes daily or occasionally. Prevalent cardiovascular diseases were defined as any coronary heart disease (CHD) (myocardial infarction or angiographic proven CHD) and cerebrovascular events (ischemic or hemorrhagic stroke) [22, 23]. Diabetes mellitus was defined as a fasting plasma glucose ≥126 mg/dl or use of anti-hyperglycemic agents.

### Framingham hypertension risk score prediction model

Framingham hypertension risk score was derived from 1717 individuals (54% women), aged 20 to 69 years old, who were free of hypertension, cardiovascular diseases, and diabetes at the time of the baseline examination of the Framingham Offspring Study in 1979 followed to 2001. A Weibull regression model was computed along with covariates of age, BMI, SBP, and DBP as continuous variables, as well as sex (women vs. men), smoking (current vs. former or never smoker), and parental history of hypertension (both, one, or no parental history) as categorical variables, and an interaction term between age and DBP. The predicted risk of hypertension was calculated for each participant using the below equation:

$$ \hat{p}=1-\exp \left(-\mathit{\exp}\left(\frac{\ln (t)-\sum \limits_{i=0}^p{\beta}_i{X}_i}{\sigma}\right)\right) $$

Where t = time in years between examinations, *β*_*i*_ = the regression coefficients of interested covariates and σ = scale parameter. The values of the coefficients and definitions of covariates are in Supplementary Table S1.

### Statistical analysis

The 5423 Participants were followed a single period from examination cycle five to six, contributing to a total of 12,855 person-years at risk. We examined the validity of the Framingham risk score in four stages [21]. First, we calculated the Framingham risk score using the *β*-coefficients derived in the Framingham study. Second, we recalibrated the Framingham risk score by updating the intercept; we replaced the intercept and scale parameter of the Framingham risk score with those of the TLGS, considering the linear predictor based on the original model as the offset in the model. Third, we recalibrated the Framingham risk score by another simple updating approach; we updated the intercept and calibration slope, considering the linear predictor as the only covariate in the model. Fourth, we revised the Framingham risk score by a more extensive updating approach (model revision), recalibration, and re-estimation of the coefficient of the sex covariate by fitting a Weibull model, in which the linear predictor and sex are the only covariates. This modeling choice was motivated by a difference between TLGS and Framingham regarding the hazard ratio of sex.

We assessed the performance of the Framingham risk prediction model among the TLGS population according to three evaluations: equality of regression coefficients (hazard ratio, HR); discrimination; and calibration. To compare the coefficients between the TLGS study and the Framingham study, we used a Weibull model using the same covariates in the Framingham model. A Z test statistic was calculated as:

$$ Z=\frac{\left({\beta}_F-{\beta}_T\right)}{\sqrt{\Big({SE}_F^2}-{SE}_T^2\Big)} $$

Where *β*_*F*_ and *β*_*T*_ are the regression coefficients of the Framingham study and the TLGS, respectively, and ${SE}_F^2$ and ${SE}_T^2$ are the squares of the SEs for the two coefficients [25]. Next, we assessed discrimination based on Harrell’s concordance statistic (c-index). For the internal validation of the model updating, the revised model’s performance was also evaluated by assessing the distribution of the c-indexes in 1000 bootstrap samples derived from the original data set with replacement [26]. Calibration included comparing the predicted hypertension incidence with the observed incidence for each decile of the risk score in a graphical assessment (calibration plot). The ratio of the predicted to observed risks was also calculated for the whole validation cohort. Furthermore, an additional analysis was conducted by including individuals with diabetes. All of the analyses were done with Stata version 14.2 (StataCorp. 2015. College Station, TX: StataCorp LP.).

## Results

### Baseline characteristics

Table 1 shows the baseline characteristics of the 5423 participants. The mean age of the participants was 38.7 years, and 56.6% were women. Mean systolic and diastolic blood pressure was 108.8 and 73.8 mmHg, respectively; approximately 26% of participants had a history of hypertension in at least one parent. Between the 5th and 6th examination cycles (median, 3.04 years), 319 persons (176 men) developed new-onset hypertension. The incidence rate of hypertension (per 1000 person-years) was 24.8 (95% CI, 22.2–27.7).

### Comparison between the Framingham and TLGS models

Table 2 shows the coefficients and hazard ratios for the incidence of hypertension in the TLGS in comparison to those of the Framingham Weibull model. The hazard ratio for women versus men was significantly different in the TLGS, compared to that obtained in the Framingham study (0.809 versus 1.260). Besides, the hazard ratio for systolic blood pressure was slightly smaller than that in the Framingham study (1.052 versus 1.070). Still, not all of the other hazard ratios were significantly different between the present study and the Framingham study.

**Table 2:** Hazard Ratios of Risk Factors for Incident Hypertension in the TLGS and Framingham Study

Parameter/Predictor	TLGS Hazard Ratio (95% CI)	Framingham Hazard Ratio (95% CI)	***P***-value *
**Age (per year)**	1.22 (1.08–1.38)	1.20 (1.09–1.31)	0.72
**Women (vs men)**	0.81 (0.63–1.04)	1.26 (1.09–1.46)	0.006
**Systolic blood pressure (per 1 mmHg)**	1.05 (1.04–1.07)	1.07 (1.06–1.08)	0.03
**Diastolic blood pressure (per 1 mmHg)**	1.19 (1.10–1.29)	1.16 (1.09–1.23)	0.74
**Current smoking (vs not)**	1.35 (0.98–1.86)	1.24 (1.06–1.46)	0.79
**Parental hypertension (vs not)**	1.43 (1.17–1.82)	1.209 (1.05–1.40)	0.66
**Body mass index (per unit)**	1.04 (1.02–1.07)	1.039 (1.03–1.05)	0.74
**Age by diastolic blood pressure**	0.99 (0 .99–0.99)	0.99 (0.99–0.99)	0.74

*Data show the *P*-value for the difference in β-Coefficient between this study and the Framingham study [12]

### Performance of the Framingham hypertension risk score

**Fig. 1:**
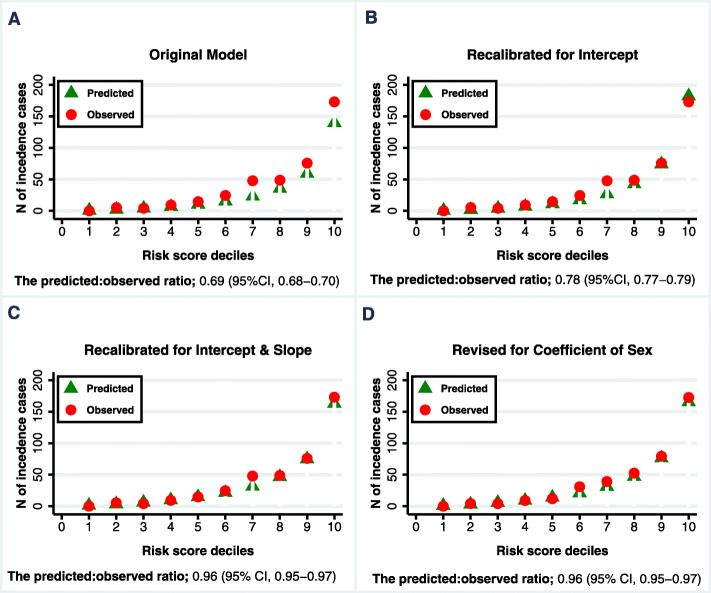
The Number of Predicted and Observed Incident Hypertension Cases by Deciles of the Original (**a**), Intercept Recalibrated (**b**), Slope Recalibrated (**c**), and Revised (**d**) Framingham Risk Scores

Table 3 shows the ratios of predicted to observed hypertension incidence in each decile of the predicted risk. The original Framingham risk score underestimated the observed hypertension risk within each decile; however, recalibration and model revision improved the performance of the model (Fig. 1). The ratio of the predicted to observed risks was 0.69 (95% CI, 0.68–0.70)) for the original Framingham risk score and 0.96 (95% CI, 0.95–0.97) for the revised risk score. The c-index was 0.81 (95% CI: 0.79–0.83) for the original Framingham risk score and 0.82 (95% CI: 0.80–0.84) for the revised risk score. The bootstrap bias-corrected c-index was 0.82 (95% CI: 0.79 to 0.83), indicating a stable predictive capability.

**Table 3:** Ratios of Predicted to Observed Hypertension Incidence in each Decile of the Risk Predicted by the Framingham Risk Score

Deciles		1	2	3	4	5	6	7	8	9	10
**Original model**	Predicted, n^a^	0.8	2.2	4.1	6.7	10.3	15.4	23.1	35.4	59.3	139.8
	Observed, n^b^	0	5.4	4.2	9.3	14.7	24.5	47.8	48.7	75.9	173.1
	Ratio P: O	–	0.41	0.97	0.72	0.72	0.63	0.48	0.73	0.78	.081
**Recalibrated for intercept**	Predicted, n	0.69	2.1	4.0	6.9	11.1	17.2	26.8	42.6	74.3	182.3
	Observed, n	0	4.2	4.2	9.3	14.7	24.5	47.8	48.7	75.9	173.1
	Ratio P: O	–	0.38	0.96	0.75	0.76	0.70	056	0.87	0.98	1.05
**Recalibrated for intercept & slop**	Predicted, n	1.3	3.5	6.2	9.9	14.8	21.6	31.5	46.7	75.0	163.0
	Observed, n	0	5.4	4.2	9.3	14.7	24.5	47.8	48.7	75.9	173.1
	Ratio P: O	–	0.65	1.5	1.07	1.01	0.88	0.66	0.96	0.99	0.94
**Revised for the coefficient of sex**	Predicted, n	1.1	3.1	5.8	9.2	14.0	21.0	31.3	47.2	76.3	165.4
	Observed, n	0	4.2	4.1	9.0	11.9	30.9	39.2	52.5	79.2	172.7
	Ratio P: O	–	0.73	1.4	1.02	1.2	0.68	0.80	0.90	0.96	0.96

*n* number; *P* predicted numbers; *O* observed numbers

^a^The average number of predicted hypertension cases in each decile

^b^The average number of observed hypertension cases in each decile

### Including individuals with diabetes

At baseline (examination cycle 5), 316 individuals had diabetes, 52 of whom developed new-onset hypertension during follow-up. Adding these diabetic subjects to the study population resulted in a c-index of 0.81 (95% CI: 0.79, 0.84) for the FHS model and 0.82 (95% CI: 0.80, 0.84) for the revised model.

## Discussion

In this population-based cohort study of non-hypertensive adults aged 20 to 69 years, we applied the Framingham hypertension risk function to predict the 3-year absolute risk of incident hypertension. In the TLGS population, HRs of risk factors for incident hypertension events were significantly similar to those obtained in the Framingham study. The only difference of potential importance that we noticed was a different result in the contribution of sex to the risk of hypertension and a slightly lower hazard ratio for systolic blood pressure.

Our study, in contrast to the Framingham study [10], showed that women were less likely to be hypertensive compared to men (HR = 0.809). In line with our study findings, some previous studies demonstrated that among individuals with the same age until the sixth decade of life, men have a higher incidence of hypertension compared to women [24, 27–29]. Sex differences can be attributed to biological and behavioral factors [30]. Although the biological differences between men and women are the same in the two communities of the TLGS and Framingham, behavioral factors, including smoking, physical activity, alcohol consumption, and other culturally related behaviors (e.g. due to religious beliefs) are different [31, 32]. For example, Iranian women smoke less and consume less alcohol, and are less educated and more likely housekeeper. These behaviors may protect them against hypertension.

In the current study, we showed that the Framingham hypertension risk score has a high ability to discriminate individuals who developed hypertension and those did not in the TLGS cohort (c-index = 0.82). This risk score systematically underestimated the risk of hypertension; however, it was able to be corrected by the process of recalibration and model revision. We indicated that both recalibrated and revised models have proper calibration for predicting the risk of incident hypertension. The ratio of the predicted to observed risks across the entire score deciles also confirmed the improvement of the revised Framingham risk score. (The ratio improved from 0.69 (95% CI, 0.68–0.70)) for the original Framingham risk score to 0.96 (95% CI, 0.95–0.97) for the revised risk score without any overlap between CIs).

We did recalibration as the first and the most straightforward step of updating a prediction model in a new population to address systematic over-or under-estimation of the risk [21, 25]. We also did model revision since it is a more complicated and extensive approach to updating a prediction model to modify the equation for differences in baseline incidence and the associations between the outcome and risk factors [21]. In this way, we addressed the significantly different HR for sex in the TLGS compared to that in the Framingham population. This point affected the performance of the Framingham prediction model in our community slightly.

Given that current ADA guidelines recommend a BP goal of < 140/90 mmHg for most patients with diabetes [33], we also assessed the validity of the Framingham risk score by including individuals with diabetes; however, the validity findings provided no marked difference.

The predictive performance of the Framingham hypertension prediction model has been tested on different populations. Consistent with our findings, the results from the MESA study showed that the Framingham risk score provides good discrimination but underestimates the risk of incident hypertension in some ethnic groups. Still, it could be corrected using a recalibration process [34]. Also, the performance of the Framingham risk prediction model was assessed in a younger population (age 18–30 years); the model in the CARDIA population performed well but systematically underestimated the risk [35]. In contrast, the 5-year predictive ability of the Framingham risk score in the Whitehall II study was reasonable, given both calibration and discrimination, but slightly overestimated hypertension risk among individuals < 50 years old. They showed that reclassification based on the Whitehall model, i.e., the model with the same variables in the Framingham but new beta coefficients in the Whitehall II population, does not improve the prediction [36].

Bozorgmanesh, et al. have developed a point-score system for predicting incident hypertension in the TLGS study [37]. The c-index for this prediction model was 0.73 among women and 0.74 among men. This is substantially lower than found for the Framingham model in our evaluation. A reason may be that their model did not include the family history of hypertension as a well-known predictor for incident hypertension [12].

It has been demonstrated that a targeted preventive strategy in individuals at high risk of developing hypertension is an effective strategy for the prevention of hypertension [38, 39].

Prediction models for CVDs, e.g. WHO CVD risk scores, are planned to be routinely used in primary health care using data on routinely measured conventional risk factors. Since these data are common to the hypertension prediction model, joining CVD and hypertension risk predictions in primary care can be an opportunity at no extra cost for NCD prevention programs.

### Strengths and limitations

The strength of the current study is that it included a large population-based cohort of both sexes. This study also has several limitations. First, TLGS is comprised of urban adults in Tehran, and generalizing the results to mainly rural individuals should be done with caution. Also, generalization beyond the Middle East may be limited. Second, we defined the incidence of hypertension based on blood pressure measurements taken on a single visit, which may be less accurate than several measurements to confirm hypertension diagnosis; however, it is a common method in observational studies. Finally, like other population-based cohort studies, selection bias due to excluding missing data is a concern. We repeated all the analyses using multiple imputations and the results did not change (data not shown). However, since we followed the original study’s exclusion criteria for the Framingham model, we excluded missing covariates as they did.

## Conclusion

These data suggest that the Framingham hypertension risk score systematically underestimates the risk of hypertension; however, the process of recalibration and model revision can correct it. Our investigation represents that the revised Framingham hypertension risk score can be used as a screening tool in public health and clinical practice to facilitate the targeting of preventive interventions in high-risk Middle Eastern people.

## Supplementary Information

**Additional file 1. Table S1. **Model Parameters for Incident Hypertension in the TLGS and Framingham Study.

## Data Availability

The datasets used during the current study are available from the corresponding author on reasonable request.

## Abbreviations

TLGS: Tehran Lipid and Glucose Study
C-index: Harrell’s concordance statistic
GBD: Global Burden of Disease
SBP: Systolic Blood Pressure
DBP: Diastolic Blood Pressure
DALY: Disability-Adjusted Life Year
NCD: Non-Communicable Disease
BMI: Body Mass Index
CHD: Coronary Heart Disease
HR: Hazard Ratio
ADA: The American Diabetes Association
MESA: Multi-Ethnic Study of Atherosclerosis
CARDIA: The Coronary Artery Risk Development in Young Adults Study
