# Supplementary material for: Validation of the Framingham hypertension risk score in a middle eastern population: Tehran lipid and glucose study (TLGS)
Source: BMC Public Health. 2021 Apr 24;21:790. doi: 10.1186/s12889-021-10760-6 (PMC8070324; doi:10.1186/s12889-021-10760-6)
Supplement: Supplementary file 1 — Additional file 1. Table S1. Model Parameters for Incident Hypertension in the TLGS and Framingham Study. [file 12889_2021_10760_MOESM1_ESM.docx]

**Validation of the Framingham hypertension risk score in a middle eastern population: Tehran lipid and glucose study (TLGS)**

Fatemeh koohi^1,2^, Ewout W. Steyerberg^3^ , Leila Cheraghi^2^, Alireza Abdshah^4^, Fereidoun Azizi^5^, Davood Khalili^2,6*^

1. Student Research Committee, Department of Epidemiology, School of Public Health and Safety, Shahid Beheshti University of Medical Sciences, Tehran, Iran.
2. Department of Epidemiology and Biostatistics, Research Institute for Endocrine Sciences, Shahid Beheshti University of Medical Sciences, Tehran, Iran.
3. Department of Biomedical Data Sciences, Medical Statistics, and Medical Decision Making, Leiden University Medical Centre, Leiden, the Netherlands.
4. School of Medicine, Tehran University of Medical Sciences, Tehran, Iran.
5. Endocrine Research Center, Research Institute for Endocrine Sciences, Shahid Beheshti University of Medical Sciences, Tehran, Iran.
6. Prevention of Metabolic Disorders Research Center, Research Institute for Endocrine Sciences, Shahid Beheshti University of Medical Sciences, Tehran, Iran.

***Corresponding author:**

Davood Khalili, MD MPH PhD , Department of Epidemiology and Biostatistics, Research Institute for Endocrine Sciences, Shahid Beheshti University of Medical Sciences, Tehran, Iran.

Email address: [dkhalili@endocrine.ac.ir](mailto:dkhalili@endocrine.ac.ir)

**Additional file 1: Table S1**

**Table S1.** Model Parameters for Incident Hypertension in the TLGS and Framingham Study

| **Parameter/Predictor** | **TLGS**  **β-Coefficient**  **(±SE)** | **P-value** | **Framingham**  **β-Coefficient***  **(±SE)** | **P-value*** |
| --- | --- | --- | --- | --- |
| **Intercept** | 17.0917 | **-** | 22.94954 | **-** |
| **Age (per year)** | -0.1279 ± 0.0399 | 0.001 | - 0.15641 ± 0.0474 | <0.001 |
| **Women (vs men)** | 0.1355 ± 0.0809 | 0.094 | - 0.20293 ± 0.0709 | 0.004 |
| **Systolic blood pressure (per 1 mm Hg)** | -0.0322 ± 0.0046 | <0.001 | - 0.05933 ± 0.0093 | <0.001 |
| **Diastolic blood pressure (per 1 mm Hg)** | -0.1113 ± 0.0259 | <0.001 | - 0.12847 ± 0.0338 | <0.001 |
| **Current smoking (vs not)** | -0.1912 ± 0.1049 | 0.068 | - 0.19073 ± 0.0766 | 0.013 |
| **Parental hypertension‡** | -0.2302 ± 0.0796 | 0.004 | - 0.16612 ± 0.0673 | 0.014 |
| **Body mass index (per unit)** | -0.0294 ± 0.0084 | <0.001 | - 0.03388 ± 0.0078 | <0.001 |
| **Age by diastolic blood pressure** | 0.0013 ± 0.0005 | 0.008 | 0.00162 ± 0.0006 | 0.005 |
| **Scale parameter** | 0 .6401 ± 0.0333 |  | 0.87692 | NA |

NA indicates not applicable.

*Data show the β-Coefficients and P-values that were reported in the Framingham study [1].

‡ β-Coefficient for parental hypertension versus no parental hypertension as the reference category in the TLGS study and each category increment in parental hypertension (1 parent or both parents) versus no parental hypertension in the Framingham study [1].

**Reference:**

1. Parikh NI. et al. A risk score for predicting near-term incidence of hypertension: the Framingham Heart Study. Annals of internal medicine. 2008. 148**,** 102-10.
